# Immune Defects in the Risk of Infection and Response to Vaccination in Monoclonal Gammopathy of Undetermined Significance and Multiple Myeloma

**DOI:** 10.3389/fimmu.2014.00257

**Published:** 2014-06-03

**Authors:** Sarah M. Tete, Marc Bijl, Surinder S. Sahota, Nicolaas A. Bos

**Affiliations:** ^1^Department of Rheumatology and Clinical Immunology, University of Groningen, University Medical Center Groningen, Groningen, Netherlands; ^2^Cancer Sciences Unit, Faculty of Medicine, University of Southampton, Southampton, UK; ^3^Department of Internal Medicine and Rheumatology, Martini Hospital, Groningen, Netherlands

**Keywords:** MGUS, multiple myeloma, infections, immune defects, vaccination

## Abstract

The plasma cell proliferative disorders monoclonal gammopathy of undetermined significance (MGUS) and malignant multiple myeloma (MM) are characterized by an accumulation of transformed clonal plasma cells in the bone marrow and production of monoclonal immunoglobulin. They typically affect an older population, with median age of diagnosis of approximately 70 years. In both disorders, there is an increased risk of infection due to the immunosuppressive effects of disease and conjointly of therapy in MM, and response to vaccination to counter infection is compromised. The underlying factors in a weakened immune response in MGUS and MM are as yet not fully understood. A confounding factor is the onset of normal aging, which quantitatively and qualitatively hampers humoral immunity to affect response to infection and vaccination. In this review, we examine the status of immune alterations in MGUS and MM and set these against normal aging immune responses. We focus primarily on quantitative and functional aspects of B-cell immunity. Furthermore, we review the current knowledge relating to susceptibility to infectious disease in MGUS and MM, and how efficacy of conventional vaccination is affected by proliferative disease-related and therapy-related factors.

## Introduction

Understanding immunosenescence has now emerged as a priority to counter immunological susceptibility to disease in normal aging. Evidence is revealing that the B-cell compartment and the associated immune synapse in the aging immune response underlie an increased susceptibility to infections and decreased response to vaccination (1). In age-related immunosenescence, onset of hematological disease may further ameliorate immune capacity, and a combination of these factors has as yet received little attention. To address this, we focus on a non-malignant B-cell clonal expansion in late age, on monoclonal gammopathy of undetermined significance (MGUS) where plasma cells accrue to <10% of the bone marrow. This condition provides a unique model to understand how immune susceptibility is impaired in aging by disease. The model extends and acquires a further urgency when it is recognized that MGUS almost invariably precedes transformation to malignant multiple myeloma (MM) (2), in which humoral immune capacity deteriorates further. Currently however, how the efficacy of B-cell response to generate protective humoral immunity diminishes in these disease states is largely undefined. Understanding this will be crucial for successful intervention with vaccination to counter infections in MGUS and MM, and reduce the associated morbidity and mortality. Consequently, this review examines B-cell dysfunction in monoclonal disease spanning MGUS to MM.

Monoclonal gammopathy of undetermined significance incidence increases with advancing age. It is usually diagnosed by chance and is characterized by the presence of a serum monoclonal immunoglobulin (M-protein) (<30 g/L). MGUS is present in 3% of people over 50 years and up to 5% over 70 years (3, 4). A newly described subtype of MGUS in which the secreted M-protein lacks IgH to skew the free light-chain-(FLC)-ratio, defined as light-chain MGUS, occurs in approximately 0.8% of the population aged over 50 years to establish an overall prevalence of MGUS to 4.2% in persons aged 50 years and older (5, 6). MGUS requires clinical vigilance, to establish onset of MM. Seminal observations by Landgren and co-workers established that MM patients invariably had a previously recognized M-protein as MGUS during a nationwide population-based cohort screen (2). These paradigm shifting findings were substantiated by Weiss et al. by detection of a monoclonal gammopathy in prediagnostic sera in 90% of 30 myeloma patients (7). As yet, it is difficult to accurately predict the subset that will progress in MGUS after initial diagnosed and this remains an important area of investigation (3, 8). Interestingly, light-chain MGUS is also a precursor of light-chain MM, which comprises 20% of all MM cases; this association has important implications for understanding the nature of the “feeder” cell for plasma cell malignancy (9–11). Furthermore, progression rates differ, as IgH MGUS transforms at the rate of 1% per year but light-chain MGUS has a progression rate of 0.3% per year, which is significantly lower (4, 6, 12). Of particular relevance to the need to understand immune capacity in disease, it has been established that loss of immune control, among other factors is implicated in malignant transformation from asymptomatic disease to MM (13–16). The immune status in MGUS and MM therefore needs to be fully understood, for therapeutic intervention to prevent progression and to counter susceptibility to infection.

In clinical diagnosis, MGUS lack the overt clinical CRAB symptoms that define MM: hypercalcemia, renal insufficiency, anemia, and bone lesions (11). In the MGUS–MM transition, key stages of high clinical relevance can also be defined, as asymptomatic smoldering MM (SMM), plateau phase disease, and active disease. SMM fulfills the diagnostic criteria for MM, with serum M-protein ≥30 g/L and/or bone marrow clonal plasma cells ≥10% however without clinical CRAB symptoms or end organ damage that dictate therapeutic intervention. Although SMM is an asymptomatic precursor of symptomatic MM, it differs from MGUS as the risk of progression to MM is much higher (10% per year) as compared to MGUS (1% per year) (11, 17). This 10-fold increased risk alone suggests that SMM differs biologically to MGUS. The SMM–MM transition is at present a focal area of research, driven by the need to understand the molecular and cellular basis of full blown clonal transformation. Although recent therapies and better supportive care have improved the survival of MM, it remains largely incurable (median survival 4–5 years) (18, 19).

## The Risk and Spectrum of Infections in MGUS and MM

It is important when assessing susceptibility to infection in MGUS and MM that healthy controls are age-matched, to obviate an aging immunosenescence and this has generally been the case in reported studies. The risk of bacteremia in MGUS is increased ~2-fold as compared to healthy controls, with an increased susceptibility to a broad range of bacterial infections that include pneumonia, osteomyelitis, septicemia, pyelonephritis, endocarditis, and meningitis (20). In viral infections, MGUS patients have an increased risk of developing influenza and herpes zoster infections at a risk comparable to that for bacterial infections (20). High monoclonal protein at diagnosis associates with higher risks of infection: MGUS patients with monoclonal protein >25 g/L at diagnosis have the highest risk of infections, and the risk is still significantly increased in MGUS patients with monoclonal protein <5 g/L as compared to healthy controls (20). M-protein levels are most likely a surrogate for clonal expansion and concomitant immunosuppression. MGUS patients have an increased mortality due to bacterial infections, with a hazard ratio of 3:4 (21).

Depressed antibody titers to a number of common infectious pathogens have been found in several conditions associated with presence of an M-protein. Serum IgG antibody levels directed to 24 different microorganisms were evaluated in Waldenstrom’s macroglobulinemia, a lymphoma subtype secreting monoclonal IgM paraprotein, and in MGUS and MM (22). Significantly depressed antibody levels to a number of antigens, particularly staphylococcal, moraxella, pneumococcal, varicella zoster, and also for fungal antigens such as *Candida* and *Aspergillus* were observed in MGUS (22). However, a significant decrease in antibody titers was also observed in WM and MM, revealing that humoral immune response to most of these pathogens is suppressed. There appears to be an increased susceptibility to infections in MGUS that worsens as disease progresses to MM, as indicated by antibody titers. The duration of antibody response and their protective value however varies between different pathogens, with some specific antibody levels that remain stable over a long time. The variability in humoral response to different pathogens indicates a requirement to carefully dissect responses to individual infectious agents in MGUS and MM.

There is clear evidence of immune dysfunction in MM that leads to vulnerability to infection, a leading cause of morbidity and mortality. Lymphocytopenia (23), hypogammaglobulinemia (24), and granulocytopenia secondary to bone marrow infiltration and therapy (25) are factors that are consistently found to increase the susceptibility of MM patients to infections. In a study of 3107 newly diagnosed MM patients in the UK Medical Research Council Trial from 1980 to 2002, infections caused 135 deaths (45%) of all deaths, occurring within 60 days of diagnosis and with two-thirds of these being attributed to pneumonia (26). The risk of infection is highest in the first 3 months and decreases with response to treatment, revealing a direct causative links as tumor burden is reduced. The most frequent infections are bacteremia and pneumonia caused by *Haemophilus influenzae, Streptococcus pneumoniae*, and *Escherichia coli* (27–29). These microorganisms predominate in the early stages of disease and in plateau phase, but in the terminal phase of the disease the spectrum of causative microorganisms widens (29, 30). Recurrent bacterial infections at presentation meet the diagnostic criteria for symptomatic MM (11).

In addition to intrinsic disease-derived factors, the type of therapy used in symptomatic MM also plays a role in susceptibility to infection. Chemotherapy can disrupt the mucosal barriers thereby increasing the risk of infections (31). Induction therapy for MM has changed recently and the traditional oral melphalan and prednisone (MP) as well as vincristine–adriamycin–dexamethasone (VAD) combinations have been replaced by dexamethasone, thalidomide, bortezomib, and lenalidomide-based regimens (32, 33). Although well- and better-tolerated, the use of novel therapies results in an increased risk of opportunistic infections as well as the shift in the spectrum of infections in MM. Novel therapeutic agents increase the risk of viral infections; bortezomib therapy for instance, increases the risks of herpes zoster reactivation in the first few months of treatment due to the immunosuppressive effects on T cells (34, 35). Dexamethasone use is associated with a greater risk of infections, and associates with depressed cell-mediated immunity against cytomegalovirus and varicella-zoster virus (36, 37). Notably, high-dose dexamethasone is associated with higher rate of infections (18%) in comparison to low-dose dexamethasone (9%), as shown in a randomized controlled trial of newly diagnosed MM (38). The lack of immune reconstitution due to poor disease response to therapy leaves patients with an on-going immune deficiency that perpetuates their risk of infections. It is also conceivable that infections may have a potential role in enhancing the survival of myeloma cells but this has as yet not been fully addressed. Infections are frequent in MM and microorganisms are known to induce B-cell activation through Toll-like receptors (TLR). MM cells express TLR and TLR-specific ligands have been shown to induce cell proliferation and prevent apoptosis of human myeloma cell lines (39, 40). This further exemplifies an unwanted tumor adaptation to exploit local niche characteristics.

## Normal Age-Associated Changes in Humoral Response

The immune status of patients with MGUS or MM has to be seen in the light of the aging immune system. Qualitative as well as quantitative changes in the humoral immune response occur with late age. The B-cell repertoire and maturation response are critical in mediating protection against infection (41). Antigen response in B cells may occur with T-cell help for T-cell-dependent (TD) maturation, or can result in T-cell-independent (TI) antibody formation. Both can generate B-cell memory, although the TD pathway is largely responsible for long-term memory by generating plasma cells that home to and reside in the bone marrow; both pathways are a pre-requisite in vaccination aimed at inducing protective antibody titers (42).

A number of studies have shown a decline in total CD19+ B cells frequencies with age, and shifts in specific B-cell subset populations (43–45). A decrease in naïve B cells together with memory and plasma cells has been reported (46). In B-cell memory, the IgM component (CD19+ IgM+ IgD+ CD27+) plays an important role in response to bacterial infections and declines with age (47). There are also age-related decreases in the IgD− CD27+ switched memory B-cell pool (43, 47, 48). Furthermore, a decrease in the number and percentage of circulating plasma cells is seen with age (49). Newly arising subpopulations termed “age-associated B cells,” defined as CD21low, CD23low, CD11c+ that seem to be more responsive to TLR7 and TLR9 ligands than direct B-cell receptor (BCR) stimulation have been described (50) Antigen-specific B cells populations are altered with the changes evident as decreased antibody responses to influenza and pneumococcal vaccination (47, 51). In mechanistic insights, key observations suggests an intrinsic defect in deletional class switch recombination (CSR) due to decreased expression of activation-induced cytidine deaminase (AID) with age (43, 52). AID is a pre-requisite for both TD and TI B-cell responses. As a result, weak and low affinity antibody responses are elicited. Induction of sustainable B-cell responses is therefore a challenge in the elderly.

Changes in B-cell subpopulations impact on the available repertoire in aging and this has been mapped by spectratyping and gene sequencing studies looking at immunoglobulin heavy-chain variable region complementarity-determining region 3 (CDR3) use. These studies reveal a collapse in BCR diversity due to B-cell oligoclonal expansions that occur with age (53). Oligoclonality is also associated with poor health status (frailty) (54). This loss of diversity will clearly impair the B-cell response to new antigenic challenge.

Senescence of humoral immunity impairs immunogenicity and efficacy of vaccination with a number of studies providing evidence that age impacts on response (55–57). Immune response to influenza vaccination declines with age. In young adults, vaccination is 70–90% effective against influenza (protective titers against two or three of the influenza viral strains) especially when the vaccine matches the circulating strains (58, 59). However, in the elderly population, influenza vaccination is only 60% effective, although it may be up to 80% effective in the prevention of serious influenza complications (60, 61). Other than the inferior antibody response generated in the elderly in comparison to that of young adults (56, 62), a decreased anti-viral cell-mediated response is also apparent (56).

Infections caused by encapsulated extracellular bacteria such as *Streptococcus pneumonia* and *Haemophilus influenza* type B (Hib) are an important health issue in aging. The bacterial polysaccharide capsule *per se* provides a challenge for vaccine design to counter invasive disease. Capsular polysaccharides as antigens do not generate a memory response (63) and the antibody response to polysaccharide vaccine is short-lived (64). However, polysaccharide vaccines are more immunogenic when conjugated to a carrier protein (65, 66), as a result of the switch from a TI to a TD response. A 23-valent pneumococcal polysaccharide vaccine is currently licensed for use in the elderly since they become infected with a broad range of serotypes. Nonetheless, the effectiveness of the 23-valent pneumococcal polysaccharide vaccine in the elderly remains controversial. In a study by Rubins and colleagues, only 20% of the elderly (>65 years) had a twofold increase in specific antibody following vaccination. Moreover, they did not respond to the most prevalent serotypes causing invasive disease (67). An earlier study of elderly males showed a response to all seven measured serotypes that was comparable to that of young adults (68). Other studies have shown that vaccination reduces the incidence of hospitalization and mortality in the elderly (69–71) and is effective in 45% vaccinated individuals in preventing *Streptococcus pneumonia* infection (70). Nasopharyngeal carriage of *Streptococcus pneumonia* has the highest prevalence in infants and naturally induced anti-polysaccharide IgG prevents carriage in the adult (72). However, in the elderly naturally acquired anti-polysaccharide IgG and IgM decrease with age (73), and additional protection is not conferred by simultaneously administering pneumococcal and influenza vaccinations in the elderly (74, 75). Of the six serotypes of encapsulated *Haemophilus influenza*, type b is the most virulent, causing invasive diseases such as meningitis, septicemia, and pneumonia. A number of Hib-conjugate vaccines are licensed for use and the estimate of protective anti-Hib polysaccharide antibodies, 0.15–1 μg/mL (76, 77), are based on the assumption that protection from invasive disease is solely mediated by antibodies, with negligible contributions of cell-mediated immunity. In the elderly, Hib polysaccharide conjugate vaccine-induced specific IgG antibodies are of comparable affinity as those generated in young adults (78), and Hib polysaccharide conjugate vaccination elicits a strong and rapid response in the elderly that results in protective titers (79).

In addition to diminution of antibody responses, an increase in reactivity toward autologous antigens that leads to autoantibody production in the elderly is indicative of a wider malaise in humoral responses, suggesting loss of tolerogenic control and check-points (80–82).

Alterations in T-cell immunity that can contribute to the development of infections also occur with aging; these thymic atrophy-related changes in T-cell proportions as well as function changes have been reviewed elsewhere (83–85).

## Immune Defects in MGUS and MM

### B-cell function

B-cell dysfunction is less profound in MGUS than in MM. This can be tracked by studies on uninvolved polyclonal serum immunoglobulins as hypogammaglobulinemia, which reflects the influence of the tumor clone on the spectrum of immunoglobulin production. Hypogammaglobulinemia occurs at a lower frequency in MGUS (25–28%) (3, 86, 87), is more frequent in SMM (45–83%) (17, 88, 89) and is often associated with MM (25, 90). The frequency of hypogammaglobulinemia associates with disease progression and the reciprocal depression of uninvolved immunoglobulins is a prognostic factor for progression to MM (17, 88, 89, 91, 92). These findings support the concept that progressive immunodeficiency is a feature of disease progression in MM.

#### B-cell enumeration

Circulating CD19+ B cells in MGUS are numerically normal or decreased in comparison to age-matched HCs (93–95). In MM, the significant depression of circulating CD19+ B-cell frequencies correlates inversely with stage of disease, and coupled with defective cell-mediated immunity in late stage disease contributes to the increased risk of infection (25).

Plasma cells are the long-term depots of antibody generating cells in the normal BM. MGUS plasma cells display phenotypic heterogeneity as compared to their normal counterparts. Based on the expression of CD19 and CD56, two distinct subpopulations of plasma cells can be seen. The first population of plasma cells is polyclonal and has an identical phenotype to normal BM plasma cells, observed as CD19+ CD56− CD38+ CD138+. A second population of abnormal plasma cells is characterized by restricted intracytoplasmic light-chain expression and lack of expression of CD19 and/or CD56 [Ref. (96) – this paper does not look at CD45]. These abnormal plasma cells can be detected at low frequencies (limit of detection 0.01% of leukocytes) by using combined immunophenotyping and clonality assessment (97, 98). Abnormal MGUS clonal plasma cells have the same phenotype as MM clonal plasma cells (99). There is a progressive replacement of normal BM polyclonal plasma cells by clonal (tumor) plasma cells as disease advances from MGUS to MM (100, 101). Normal plasma cells have been found to make up to 86% of total BM plasma cells in MGUS and 0–32% in MM (96). The number of residual polyclonal plasma cells in BM can therefore distinguish between MGUS and MM with 98% MGUS patients having >3% normal plasma cells and only 1.5% MM having >3% normal plasma cells. From these observations alone, it is apparent that normal (polyclonal) long-lived plasma cell memory deteriorates markedly in MM.

Not only is the increase in aberrant clonal plasma cell populations likely to impact on normal plasma cell compartment, but also associates with egress into the circulation. The circulating abnormal plasma cells have been found to be a significant predictor of progression in MGUS (91, 100, 102, 103), and clonal plasma cells can also be detected in the peripheral blood of SMM as well as MM (104–106). Circulating abnormal plasma cells can be detected in up to 20% of MGUS patients (102, 103) and up to 80% MM (107, 108). The increase in circulating plasma cells parallels their increase in bone marrow (101).

### T-cell function

Several studies have sought to evaluate defects in T-cell frequencies and function broadly in MGUS and myeloma. At present, there is limited evidence for both decreased antigen-specific T-cell responses and less so for T-dependent B-cell antigen-specific responses.

Significant aberrations in T-cell count and function have been described in MGUS and MM. A decrease in CD4+/CD8+ ratio due to increased CD8+ T cells in the bone marrow and circulation has been reported in both conditions (95), or due to lower CD4+ T-cell numbers (102), whereas others have shown that the MGUS CD4+/CD8+ ratio and absolute numbers do not differ significantly from healthy controls (109). Within the CD4+ T-cell compartment in MM, selective loss of the naïve CD4+ CD45R+ subset has been reported (110).

Th1/Th2 cytokine balance regulates the immune response. Th1/Th2 polarization depends on the local cytokine concentrations that induce differentiation of naïve T lymphocytes to either Th1 cells, which promote cell-mediated immunity, or Th2 cells, which promote antibody-mediated immunity (111, 112). In many hematological malignancies, however, the balance is changed (113, 114). In MGUS, the balance seems comparable to healthy individuals (115), whereas in MM, there is an imbalanced Th1/Th2 ratio that results in a defective Th1 response (115). This defective Th1 response correlates with disease stage and is related to elevated levels of IL-6 and increased numbers of IL-6+CD3+ T cells. Local concentrations of IL-12 that induce differentiation of naïve T lymphocytes to a Th1 phenotype, however, are compromised by IL-10 and fail to induce polarization of activated T lymphocytes *in vivo* in MM (115).

To examine T-cell response to infectious agent antigens, Maecker et al. characterized the *in vitro* CD8+ T-cell response in HLA-A*0201+ MM patients and healthy individuals to influenza A and Epstein–Barr virus-derived immunodominant epitopes using major histocompatibility complex (MHC)/peptide tetramers (116). Following *in vitro* stimulation, 67% healthy individuals showed an increased frequency of antigen-specific T cells for both antigens while in myeloma the magnitude of the response was reduced, with <30% reacting to both viral antigens (116). These findings need to be expanded to examine antigen-specific T-cell capacity *in vivo* to vaccination to a wider spectrum of infection-related antigens. Chemotherapy aggravates the immunodeficient state of myeloma patients, resulting in significant reductions in CD4+ as well as specific reductions in CD4+ CD45RO+ cells as compared to untreated myeloma; this decrease is strongly associated with opportunistic infections (25).

The induction of effector T-cells in response to active vaccination (e.g., to counter infection) will be influenced by functional regulatory T-cells (Tregs). Treg cells play an important role in modulating immune response, maintaining immune homeostasis, and suppressing excessive immune responses where any imbalances lead to impaired immune functions. Several subsets of Tregs have been identified; naturally occurring CD4+CD25+FoxP3+ Tregs (nTregs) that are generated in the thymus, and peripherally inducible Tregs that include type I T-regulatory (Tr1) that constitutively express IL-10 and Th3 CD4+ Tregs that produce TGF-β (117–119). Furthermore, CD8+ as well as double-negative Treg (DN Treg) cells have also been described (120, 121). Tregs can regulate the effector Th1 and Th2 responses by production of soluble cytokines IL-10 and transforming growth factor-β (TGF-β) and/or contact dependent mechanisms (122, 123).

There is significant discrepancy in the literature regarding the frequency of Treg cells and their function in MGUS and MM that is probably related to the differences in techniques and markers used to identify these cells. A report by Prabhala et al. showed that the frequency of CD4+FoxP3+ Tregs in MGUS and MM is reduced and they were functionally impaired as the Tregs had significantly reduced ability to suppress T-cell proliferation (124). In contrast, Beyer et al. found a significantly increased frequency of CD4+CD25^hi^FoxP3+ Tregs in MGUS as well as treated and untreated myeloma. Using allogenic T cell stimulation, they showed that these cells also maintained their functionality as they inhibited proliferation and IFN-γ production (125). Other studies confirmed that CD4+CD25+FoxP3+ Treg cells were increased and this increase correlated with disease activity in MM (126, 127). A marked decrease in DN Tregs has also been reported in MM (126). A reduced frequency or compromised Treg activity on the other hand may be deleterious to the host, as this results in dysfunctional T cell responses and increased risk of infections (128).

### DC function

Dendritic cells (DCs) as professional antigen presenting cells play a central role in recruiting host adaptive immunity. Using blood dendritic cell antigen (BDCA) labeled DCs, plasmatoid DC (pDC) (BDCA-2+) and myeloid DC1 (mDC1) (BDCA-1+) cells were found to be suppressed in MGUS and further reduced as the disease progressed in MM (94). The reduced number of circulating mDCs and pDCs in MM are characterized by a lower expression of HLA-DR, CD40, and CD80, in addition to an impaired induction of T cell proliferation and cytokine stimulation (129, 130). The functional defects result from IL-6 inhibiting the growth of CD34+ DC progenitors and switching development and maturation of CD34+ cells from DC toward monocytic cells with phagocytic activity but with no antigen-presentation capacity (129). Enumeration of high potency CMRF44+ CD14− CD19− DCs in MM in peripheral blood approached relatively normal numbers, expressing expected levels of CD80 and CD86. They did however exhibit functional defects with reduced capacity to up-regulate CD80 and CD86 expression after CD40L and IL-2 stimuli. This stimulatory defect may result from tumor-derived TGF-β1 and IL-10 that down-regulate CD80 (131). Potentially, this can be abrogated by anti-TGF-β1 and IL-10 antibodies. IL-12 and IFN-γ can also neutralize the failure to stimulate CD80 upregulation (132). As in other immune compartments, specific therapeutic agents can impact on DC function: bortezomib, a major drug in MM, significantly impairs the immunostimulatory capacity of mDCs (133, 134). An additional consideration is that pDCs have been strikingly shown to interact with MM cells to induce tumor cell growth and survival (135). Nonetheless, DCs remain a major target for immunotherapeutic intervention in MM (136).

While DC considerations in MGUS and MM have relevance to understanding many aspects of immune response to vaccination against infection, in relation to T-dependent B-cell responses in germinal canters, it is the follicular DC specialized subset that is important in generating B-cell memory, coupled with T_FH_ cells (137). As yet, disease impact on these secondary lymphoid organ immune cell interactions has not been investigated in MGUS or MM.

### NK cell function

Natural killer cells have a role in immune response to viral infection (138, 139), and interface with adaptive immunity. They also play an important role in initial immune response to tumor cells, but as yet there is no concrete evidence for a surveillance and anti-tumor role for NK cells in response to the premalignant clone in MGUS. Increased numbers of NK cells have been described in MGUS, in newly diagnosed MM and in low tumor burden MM (140–142). NK cell function is intact in the setting of MGUS and newly diagnosed MM. However, NK cytotoxicity against MM decreases as disease progresses (140, 141, 143). The downregulation of NK cell activating receptors NKG2D, NKp30, and CD244 in MGUS and MM in the bone marrow niche but not in the circulation suggests a possible mechanism of immune escape of the tumor clone (144). MHC I molecules are critical determinants of NK cell activity. The expression levels of MHC class I polypeptide-related chain A (MICA), a soluble ligand of NKG2D is increased on plasma cells in MGUS and is strongly expressed in advancing disease in MM (145). However, the soluble NKG2D ligand, MICA, shed by MM cells as disease progresses, as a result of the upregulation of ERp5 (146) down-regulates NKG2D on effector NK cells. NKG2D levels are diminished in MM with MICA shedding, which contributes to immune suppression (146, 147). Normal to increased lytic activity of peripheral blood, NK cells have been described in MGUS but in MM with MICA shedding NK activity is decreased (146). The immunomodulatory agents thalidomide and lenalidomide have been reported to enhance NK cell cytotoxicity against MM cells (148), highlighting an aspect of current therapy that appears to potentiate immune capacity in disease, and increased NK cells are also seen in the peripheral blood of patients that respond to therapy (149).

### The immunosuppressive microenvironment of MM

A number of factors induce the immunosuppressive microenvironment that promotes tumor survival in MM and simultaneously induces immune dysfunction. Immunosuppressive factors include increased levels of IL-6, IL-10, IL-15, and TGF-β (150). They reduce anti-tumor immunity by suppressing NK cell cytotoxicity, and IL-10 and IL-6 have been implicated in DC dysfunction, as discussed above (115, 129). TGF-β also has a central role in inhibiting IL-2-induced T cell proliferation (151). Vascular endothelial growth factor (VEGF) is an important cytokine in the MM microenvironment as it not only promotes the secretion of IL-6 but also the growth and migration of MM cells (152). Specific tumor adaptations in MM cells mediate immunosuppression. Cyclooxygenase-2 (COX-2) overexpression by MM cells, which correlates with poor outcome, is also implicated in suppressing macrophage-mediated or T-cell-mediated tumor killing (153). For effective therapy against infection, a combinational approach is required: tailored vaccination together with strategies to combat the immunosuppressive MM microenvironment.

## Humoral Immune Response to Influenza, Pneumococcal, and Hib Vaccination in MM

### Vaccination following chemotherapy

Patients with MM undergoing chemotherapy are at an increased risk of contracting influenza, but it remains uncertain as to whether these patients are able to mount sufficient immune response to benefit from vaccination. Previous studies have shown heterogeneous responses to influenza vaccination in myeloma (discussed below). A comparison of studies of immune response to influenza vaccination is limited by differences in read-outs used to interpret response to vaccination. Most studies measure antibody levels by the hemagglutinin inhibition (HI) test, and responses are defined as ≥2.5-fold rise HI after vaccination. Seroconversion is defined as a ≥4-fold rise in titer and seroprotection as a titer of at least 1:40 (154, 155). Vaccination against influenza in MM is still a matter of clinical uncertainty, in part as knowledge about the protective efficacy against clinical disease (influenza) itself is limited, especially in aging. Controlled studies in MM evaluating influenza vaccination with clinical endpoints (contracting influenza and severity of infection) are lacking. Despite this clinical uncertainty, influenza vaccination is recommended in MM patients.

In a study of MM patients the results of influenza vaccination were disappointing as only 10% achieved protection against two viral strains and 19% developed protective antibody titers to all three strains in the vaccine (156). Comparable observations were noted in another study of 70 patients with hematological malignancies of which 16 were MM cases, and of the 70 only 4 responded, but none were MM, to achieve seroprotection to all 3 viral strains (157). Moreover, two doses of influenza vaccine failed to improve antibody response to influenza vaccination in patients with hematological malignancy undergoing therapy or with recently discontinued therapy as the response rate did not increase following a second booster dose of the vaccine (158, 159). Overall, the booster influenza vaccination strategy failed to increase the antibody response in immunocompromised patients.

Notwithstanding these observations, patients with hematological malignancies, even under treatment, can mount protective immune responses against influenza vaccination. Indeed, in a study of 34 patients with chronic lymphoproliferative disorders and MM, some receiving therapy, response rates were comparable to healthy controls (160). More than 60% achieved seroprotective responses to all three viral strains, and of the six patients with MM, three developed seroprotection to all three viral strains. However, these studies were limited by the low number of subjects and by patients on diverse therapy with varying dosage intensity. This precluded detection of a relation between response to vaccination and disease state and stage and also the relation between vaccination response and treatment.

Initial studies in the early 80s that utilized the 14-valent pneumococcal capsular polysaccharide vaccine showed that MM patients responded poorly to immunization. The antibody concentrations to 12 of the 14 polysaccharides in the vaccine were simultaneously measured by radioimmunoassay. Radiolabeled polysaccharides were incubated with sera and the resulting antigen–antibody complexes were precipitated and quantified with a scintillation counter as nanograms of antibody protein nitrogen/milliliter (161). The interpretation of an adequate response to the 14-valent pneumococcal vaccine was variable since the antibody concentrations needed for protection were difficult to define (162). Low levels of antibody were reported to pneumococcal capsular polysaccharides before and after vaccination in MM patients, significantly lower than those in healthy controls (163–165). Lazarus et al. showed that in MM patients before vaccination, the grand geometric mean antibody concentrations for all serotypes combined was 91 ng/mL, which is less than their presumed protective level of 215 ng antibody nitrogen/mL (165). Following vaccination, the grand geometric mean antibody concentration remained significantly lower in MM than in healthy controls (91 versus 820 ng antibody nitrogen/mL). Thirty percent of the patients had an antibody response that might be protective for half of the vaccine serotypes. Geometric mean titers >215 ng antibody nitrogen/mL developed only for serotypes 3, 18C, and 23F. Similarly, Schmid et al. observed antibody titers >200 ng antibody nitrogen/mL only for serotypes 1, 4, 18C, and 23F in myeloma patients (163). A study of 42 MM patients reported immunization responses that were inferior to those of HCs and the low anti-pneumococcal titers correlated with risk of serious infection (166). MM patients with IgG M-protein were shown to have lower antibody titers compared to those with IgA or Bence Jones MM. In line with these observations, Schmid et al. also showed that patients with IgA MM consistently responded with higher antibody titers than IgG MM and had higher increases in levels of antibodies after pneumococcal vaccination (163). These studies indicate that generation of antibodies to pneumococcal capsular polysaccharide vaccination is impaired. This may be because pneumococcal polysaccharides are particularly weakly immunogenic in the immune suppressed state of these patients. Polysaccharide vaccines are more immunogenic when conjugated to a carrier protein (discussed above). However, MM patients elicit sub-protective to protective antibody responses to Hib polysaccharide conjugate vaccination: Nix and colleagues reported that 45% of MM had protective in comparison to 97% HC while 75% of MM patients had protective titers in a study by Robertson and colleagues (156, 167). A more recent study utilizing the currently used 23-valent pneumococcal vaccine reported that the response to pneumococcal vaccination was again disappointing, with 56% having a fourfold rise in titer and 39% achieving protective antibody titers 4–6 weeks after vaccination (156). These responses to adjuvant in conjugated vaccine flag up deficiencies in pre-requisite Th responses in MM.

### Transplantation

Ablative chemotherapy followed by bone marrow transplantation generally results in an acute immunosuppression in both arms of innate and adaptive immune system that lasts for several months, resulting in a protracted functional recovery. B-cell reconstitution after hematopoietic stem cell transplantation (HSCT) is slow, with B cells reaching normal levels 4–8 months post-transplant (168, 169). Re-population of T cells is also prolonged (170), appearing as abnormal subpopulations that show an inverted CD4/CD8 ratio (171, 172). The prolonged and severe immune suppression following autologous and allogenic stem cell transplantation not only compromises immunity and pose a risk for infection but also results in poor responses to vaccination (173–175). Impaired antibody responses to influenza have been demonstrated in this setting (159, 176), however, a longer transplant to vaccination interval is associated with better serological responses. Similarly, poor responses to pneumococcal vaccination prior to transplant (177) as well as vaccination after transplant have been reported (173, 178, 179).

In a randomized Phase 1/2 study in advanced MM undergoing high-dose mephalan and autologous stem cell transplantation (ASCT), post-transplant conjugate pneumococcal vaccination resulted in no or low antibody responses and did not increase after booster vaccination (180). However, vaccination before transplant together with the adoptive transfer of T-cells early after transplant followed by booster vaccination resulted in robust and sustained antibody response as well as accelerated T-cell recovery (180). This achieved protective titers against four serotypes (6B, 14, 19F, and 23F) in 60% of cohort as compared to 18% when there was no vaccination. In another study of MM, autologous T cell transfer resulted in poor or no influenza vaccine responses unless the patient had received influenza vaccination prior to autologous T-cell collection (181). Clearly, the priming of the autologous T cells by pre-transplant vaccination most likely enhances antigen-specific antibody induction following transfer.

These studies demonstrate that a T-cell adoptive transfer can accelerates the numerical and functional recovery of CD4+ and CD8+ T-cells that may provide help in restoring humoral immunity in MM. Strategies to enhance lymphocyte recovery and function (both B- and T-cell) in a transplant-associated therapeutic intervention could further improve outcome in achieving protective serological and cellular immunity to infectious agents in MM.

A detailed summary of the many strategies that have been assessed to date in vaccination against infection in MM is compiled in Table 1.

**Table 1:** **Results of studies of the efficacy of influenza, pneumococcal, and Hib vaccination in multiple myeloma**.

Vaccine	Study	Study design number of patients	Myeloma treatment	Measure of efficacy	Response	Conclusion
**Influenza**	Robertson et al. (156)	MM (*n* = 48)	IFNα/chemotherapy/high-dose MP/total body radiation + autologous stem cell transplantation 6 months before	GMT, titers ≥1: 40	Poor response. 19% achieved seroprotection and 59% had no seroprotective levels to any of the three strains	Poor responses and patients are susceptible to infections with influenza
	Rapezzi et al. (160)	MM (*n* = 6) CLL (*n* = 13) NHL (*n* = 7) HD (*n* = 8)	MP + prednisone/MP + prednisone + VAD	GMT, titers ≥1: 40	Seroprotection rates achieved by more than 60%. Of the patients, three of six MM achieved seroprotection rates	Vaccination is well-tolerated and safe in CLPD and MM
	Stadtmauer et al. (181)	MM (*n* = 21)	High-dose MP + autologous stem cell transplantation	GMT, ≥4-fold rise in titers	Primed subjects had significantly higher GMT at all times	Transfer of influenza-primed autologous T cells after transplantation improves subsequent vaccine responses
					73% of primed subjects had seroconversion to any of the three vaccine strains and only 30% of unprimed subjects	
**Pneumococcal**	Lazarus et al. (165)	MM (*n* = 13)	BCNU + adriamycin/MP + prednisone/cyclophosphamide/BCNU + prednisone + cyclophosphamide/MP + adriamycin + vincristine	GMT, ≥2-fold rise in titer	Poor response. 30% achieved protective response to six or more serotypes	Antibody response is depressed. Advisable to vaccinate patients as response was highly variable
	Schmid et al. (163)	MM (*n* = 37) HC (*n* = 10)	MP + prednisone/vincristine + cyclophosphamide + prednisone (and doxorubicin/MP), or another combination of three or more/no chemotherapy for at least 3 months before vaccination	GMT ≥2-fold rise in titers	At least twofold increase in titers to at least eight antigens in 43% compared to 100% HC. Poorer response in those receiving multi-agent (≥3) chemotherapy	Very low antibody titers before and after vaccination but as response was heterogeneous vaccination can be offered
	Hargreaves et al. (166)	MM (*n* = 41) HC (*n* = 62)	MP/MP + adriamycin + BCNU + cyclophosphamide/vincristine + cyclophosphamide + MP + prednisone/vincristine + BCNU + adriamycin + prednisone/vincristine + adriamycin + dexamethasone/vincristine + adriamycin + MP + prednisone	GMT, ≥2-fold rise in titer	Poor response 45% achieved protective titers	Poor response associated with increased risk of septicemia
	Robertson et al. (156)	MM (*n* = 48)	IFNα/chemotherapy/high-dose MP/total body radiation + autologous stem cell transplantation 6 months before	GMT titers ≥1:640	39% Achieved protective titers	Poor responses, likely to be poorly sustained. Repeat vaccination is desirable
	Rapoport et al. (180)	MM (*n* = 42)	High-dose MP + autologous stem cell transplant	GMT	60% of pre-transplant vaccination+ post-transplant T cell infusion recipients achieved protective titers.	Early adoptive T cell transfer followed by post-transplant booster immunization improves immunodeficiency. Pre-transplant vaccination regime superior to post-transplant vaccination regime
					18% of post-transplant vaccine & pre-transplant+ late T cell infusion recipients achieved protective titers	
	Hinge et al. (177)	MM (*n* = 60)	High-dose MP + autologous stem cell transplantation	GMT titers ≥1: 40	Poor response. 33% responded	Reasonable to vaccinate patients with disease control (responding well to induction therapy) as they have higher response rate
**Hib**	Robertson et al. (156)	MM (*n* = 46)	IFNá/chemotherapy/high-dose MP/total body radiation + autologous stem cell transplantation 6 months before	≥1.02 μg/L	75% protective titers and 41% had a ≥4-fold increase in titers	Specific immunity comparable to HC.
	Nix et al. (167)	MM (*n* = 20)	Intermittent chemotherapy	>0.15 μg/mL	45% MM achieved titers that correlate to natural protection in comparison to 97% HC	Lack of protective immunity against Hib in MM. Increased risk of invasive disease is a rationale for immunization
		Chronic renal failure (*n* = 59)	
		Diabetes mellitus (*n* = 30)	
		HC (*n* = 32)	

*CLPD, chronic lymphoproliferative disorders; HC, healthy controls; HD, Hodgkin disease; MP, mephalan; NHL, non-Hodgkin lymphoma; VAD, vincristine–adriamycin–dexamethasone*.

## Conclusion and Future Directions

Immunosenescence in aging compounds immune function in MGUS and MM, which incrementally deteriorates as disease progresses to symptomatic phase. Focusing on humoral immunity, as this is a pre-requisite to counter infection in these disease settings, it is evident that a complex array of factors underlies a diminution of immune capacity. This is manifest as both numerical imbalances in B-cell and T-cell populations, and in impaired lymphocyte functionality; the immunological synapse and cross-talk between these two key immune players is not fully defined yet in MGUS or MM, and remains a key area for further investigation. Clonal expansions in MGUS and MM directly compete with niche space for long-term memory of infection, affecting polyclonal normal plasma cells in the bone marrow. Direct immunosuppression by tumor cells, including via cytokine imbalance and other mechanisms such as aberrant ligand expression to block NK cell activity, further affect the capacity of the immune system to mount effective challenge to infection, or to vaccination aimed at enhancing immunity to both viral and bacterial agents. Because of the morbidity and mortality that follow infection in MM, clinical therapy still aims at vaccinating against specific bacterial threats. Mounting effective immunity to capsular polysaccharide infection-related antigens for instance, has as yet not been optimized although linking antigen to T-cell recruiting adjuvant is a notable advance. The complications generated directly by therapeutic drugs that weaken the immune response must be understood further, to select the best strategy to restore anti-infection immunity in MM. Conversely, use of immunomodulatory drugs in MM will need further investigation in how to best harness their pro-immune function during vaccination against infection. Exploiting transplantation in therapy by prior vaccination to educate T-cells to enhance humoral response to vaccination is indicative of the types of strategy that can be utilized to counter infection. It is also highly likely that current advances in understanding how aging in the normal healthy population affects immune responses will be exploited in developing the best strategies to institute immunity against infection in MGUS and MM.

## Conflict of Interest Statement

The authors declare that the research was conducted in the absence of any commercial or financial relationships that could be construed as a potential conflict of interest.
